# Feasibility and efficiency of delayed ovarian stimulation and metaphase II oocyte banking for fertility preservation and childbearing desire after fertility-impairing treatment

**DOI:** 10.1038/s41598-023-42583-3

**Published:** 2023-09-20

**Authors:** Laura Miquel, Julie Liotta, Alice Hours, Pauline Bottin, Pierre Castel, Jeanne Perrin, Catherine Guillemain, Blandine Courbiere

**Affiliations:** 1 Department of Obstetrics, Gynecology and Reproductive Medicine, Pôle femmes parents enfants, AP-HM, La Conception University Hospital, Marseille, France; 2 Aix Marseille Univ, CNRS, IRD, Avignon Université, IMBE, 13397 Marseille, France; 3 Aix Marseille Univ, INSERM, MMG, UMR_S 1251, Marseille, France

**Keywords:** Ovary, Quality of life, Cancer, Infertility

## Abstract

The aim of our study was to evaluate the feasibility and efficiency of delayed ovarian stimulation and metaphase II oocyte banking for fertility preservation after fertility-impairing treatment regardless of the initial disease. We conducted a cohort study based on population of women < 40 years of age with diminished ovarian reserve caused by fertility-impairing treatment (n = 129). Three groups of women were compared according to the type of initial disease: hematological malignancies, solid tumors, and benign diseases. The primary endpoint was the number of metaphase II oocytes collected per woman. We studied the cumulative live-birth rate per cycle with fertilized metaphase II oocyte, for women who wanted to conceive. We studied 245 delayed controlled ovarian stimulation cycles in 129 women: 201 for fertility preservation and 44 for in vitro fertilization and fresh embryo transfers. The number of metaphase II oocytes collected per woman after banking was similar in the three groups, with a mean of 10.7 ± 4.6, 12.3 ± 9.1, and 10.1 ± 7.6 metaphase II oocytes (p = 0.46), respectively. In the subgroup of women who wanted to conceive, the cumulative live birth rate per woman was 38%, with 8 live births for these 21 women. After fertility-impairing treatment, practitioners should discuss a fertility preservation procedure for banking metaphase II oocytes.

## Introduction

Ovarian reserve is a term used to describe the functional potential of the ovary and reflects the number and quality of oocytes within it^1^. Since ovarian reserve diminishes progressively throughout the reproductive life span until menopause, any iatrogenic damage to the ovarian reserve may lead to premature ovarian insufficiency (POI) and infertility^2^. Patients facing treatments likely to impair reproductive function deserve prompt counseling regarding their options for fertility preservation and rapid referral to an appropriate program^3^. Emergency fertility preservation (FP) of germinal tissue or metaphase II oocytes should be proposed when benign or malignant disease or therapy is likely to impair ovarian reserve^4^. Germinal tissue cryopreservation is the only option for girls with cancer at the prepubertal stage since the potential risk of stimulation of estrogen-sensitive cancer can be bypassed^5^. Since the development of vitrification, metaphase II oocyte cryopreservation is the method advised for most women undergoing FP for medical indications^6^.

Unfortunately, for various reasons (not enough time between diagnosis and the initiation of urgent cancer therapy, refusal of a woman to undergo additional therapy, emergency adnexectomy, lack to referral to FP center, etc.), emergency FP before definitive impairment of the ovarian reserve is not always possible, as for example, for borderline ovarian tumors^7^. When ovarian stimulation for fertility preservation has been performed on emergency, only a single controlled ovarian stimulation (COS) is authorized^3^. This single COS rarely yields enough metaphase II oocytes to give a chance of later pregnancy^8^. When metaphase II oocyte cryopreservation is performed before 35 years of age and before cancer treatment, the cumulative live birth rate (CLBR) is only 9.1% (CI − 0.7 to 19) if 5 metaphase II oocytes are cryopreserved and 35.8% (CI 4.3–57.2) if 8 metaphase II oocytes are cryopreserved^9^. As illustrated by the case of lymphoma, the number of cryopreserved metaphase II oocytes is generally lower than that in cases of other malignant diseases^10^**.** In this case, delayed FP performed after chemotherapy treatment and remission could allow metaphase II oocyte banking to increase the probability of live birth after metaphase II oocyte thawing^11^. Offering the possibility of delayed FP after fertility-impairing treatment when emergency FP cannot be achieved is sometimes the only option. Starting stimulation before the occurrence of POI becomes a necessity to offer these women a chance to build a family. When due to a malignant disease, alteration in female fertility has an impact on women's quality of life^12^. Evaluated the benefit of FP on quality of life in women with benign diseases is still a challenge. This systematic delayed FP strategy for women with diminished ovarian reserve (DOR) was implemented in 2015 our fertility preservation unit^13^.

The objective of our study was to investigate the feasibility and efficacy of a center-based strategy for delayed FP after fertility-impairing treatment regardless of its cause.

## Materials and methods

### Study population

We conducted a retrospective cohort study in a French fertility preservation unit of a university teaching hospital between January 2015 and December 2020. The study protocol was approved by the ethics committee of the Aix-Marseille University on September 8, 2021 under the number 2021-09-07-03. The research was performed in accordance with relevant regulations, and informed consent was obtained from all participants.

We included all reproductive-age women referred for counseling after fertility-impairing treatment for malign or benign pathologies. Women presented either for a first delayed FP after fertility-impairing treatment or as an additional cycle when a first emergency FP could be performed before fertility-impairing treatment. The inclusion criteria were as follows: women < 40 years of age with a history of fertility-impairing treatment (ovarian surgery, pelvic radiotherapy, total body irradiation, history of gonadotoxic treatment), regardless of the stage of life at which exposure to fertility-impairing treatment occurred, and presenting with DOR (an abnormal ovarian reserve test (i.e. AFC < 5–7 follicles or AMH < 0.5–1.1 ng/ml)^14^. The noninclusion criteria were as follows: women with permanent amenorrhea and POI (postmenopausal levels of FSH (> 40 IU/L), four or more months of secondary amenorrhea, and age < 40 years) and women who refused to have their medical records used for the investigation^15^.

The following three groups of women were compared according to the initial disease that led to fertility impairment:

- Hematological malignancies group: Women who had undergone COS after chemotherapy or radiotherapy for hematological malignancies: mature lymphoid neoplasms (B-cell, T-cell, and Hodgkin lymphomas); diffuse large B-cell lymphoma; acute myeloid leukemia; acute lymphoblastic leukemia; Burkitt lymphoma.
- Solid tumors group: women who had undergone COS after chemotherapy or radiotherapy for solid tumors: breast cancer; ovarian cancer; nephroblastoma; rectal cancer; Ewing's sarcoma; osteosarcoma; gastric cancer; peritoneal pseudomyxoma; astrocytic glioma.
- Benign diseases group: women who had undergone COS after benign gynecological ovarian surgery or gonadotoxic medical therapy for noncancerous pathologies: ovarian endometriosis; ovarian cyst; ovarian torsion; systemic lupus erythematosus; histiocytosis; Thalassemia major with hemochromatosis.

All these diseases and their treatment are considered to impair fertility in women and are a valid indication for medical female fertility preservation^16^.

### COS protocol and metaphase II oocyte vitrification/warming

The COS protocol was prescribed as usual for the three groups, at least 2 years after the end of a mutagenic risk treatment and after the agreement of the woman's treating oncologist when necessary^17^.

The choice of the FSH starting dose was made in accordance with clinical history and the ovarian response to stimulation in previous IVF cycles. If no previous cycles have been performed, the choice have been based on such criteria as women’s age and markers of ovarian reserve^18^.

All women were monitored with serial transvaginal ultrasounds and hormonal dosages were monitored during stimulation. Ovulation was triggered either by subcutaneous HCG (Ovitrelle, Merck Serono, Germany), by 0.3 mg of triptoreline (Decapeptyl, Ipsen, France) or by both at the same time (dual triggering). The oocytes were retrieved 36 h later in the operating room and taken to the laboratory for vitrification. One hour after egg collection, the oocytes were denuded, and their maturity was assessed (presence of a polar cell).

Only metaphase II oocytes were vitrified before the 38^th^ hour after triggering. When the number of vitrified metaphase II oocytes at D0 was < 8 and when immature oocytes remained prolonged for another 24 h. Oocyte maturity was then reassessed and newly metaphase II oocytes were vitrified at D1; nevertheless, the optimal timing was not respected for these metaphase II oocytes, as they were vitrified > 38 h post triggering.

Vitrification Freeze Kit (Vit Kit-Freeze NX, FUJIFILM Irvine Scientific, USA) and vitrification straw (CBS high-security vitrification straws, IMV Technologies, Cryo Bio System, France) were used for the procedure. When thawing, a vitrification thawing kit (Vit Kit-Thaw NX, FUJIFILM Irvine Scientific, USA) was used. The metaphase II oocytes were thawed mid-morning for microinjection in the early afternoon.

In the subgroup of women who wanted to conceive, metaphase II oocytes were fertilized in vitro according to the standard protocol of our laboratory. For the use of fresh metaphase II oocytes, ICSI was performed only when sperm abnormalities were present. Morphologically normal and motile sperm were selected, as recommended by the international guidelines^19^. ICSI was systematically performed for the fertilization of thawed metaphase II oocytes. Embryo quality was assessed 48 and 72 h after fertilization. Embryo scoring was assessed at 400× magnification by evaluating cell number, size and symmetry, percentage of fragmentation and multinucleation, as recommended by the international guidelines^19^. We transferred 1 or 2 embryos under ultrasound guidance using a flexible transfer catheter (Cook, Bloomington, USA). Supernumerary embryos were frozen for subsequent embryo transfer.

### Outcome measures

The primary endpoint was the cumulative number of metaphase II oocytes collected per woman who started COS cycle, whether vitrified or immediately used for IVF. We studied these results per stimulation cycle and per woman.

The secondary endpoints were as follows: (1) the total number of COS cycles performed per woman; (2) the COS cycle cancellation rate for absence of ovarian response; (3) the metaphase II oocyte retrieval failure rate; and (4) the CLBR per stimulation cycle with fertilized metaphase II oocyte in women who wanted to conceive, including fresh and frozen embryo transfers.

Standard definitions were used as follows: absence of ovarian response was defined by no follicular growth upper than 14mm after COS, biochemical pregnancy was defined as positive HCG blood test (> 5 mUI/mL) 15 days after embryo transfer; clinical pregnancy was defined as the visualization of a positive fetal heartbeat on ultrasound at 7 weeks of gestation; and live birth was defined as a live birth after 22 weeks of gestation according to The International Glossary on Infertility and Fertility Care^20^.

### Statistical analyses

Quantitative variables were compared using one-way ANOVA with Dunnett’s post-hoc comparison test or the corresponding nonparametric test when appropriate. Categorical variables were analyzed with the chi-square test, Fisher’s exact test or the appropriate test. Variables were analysed with the appropriate parametric or nonparametric statistical test, following the validity conditions. The statistical analysis was performed using GraphPad Prism software v.8.0.1 (GraphPad Software, La Jolla, CA, USA) with a statistical significance threshold set at 0.05.

## Results

During the study period, 8978 women of reproductive age presented to our fertility center for FP or childbearing desire. Among these 8978 women, 445 were referred for delayed FP counseling after fertility-impairing treatment for malign or benign pathologies. Among these 445 women, 69 presented with POI, 17 were too old to benefit from metaphase II oocyte cryopreservation (> 40 years), 90 had a normal ovarian reserve, 62 preferred to delay COS for metaphase II oocyte cryopreservation, and 2 refused to have their medical records used for the investigation.

At the same time 76 women did not wish to undergo COS after counseling. Forty-five women did not reply to phone calls and were therefore lost to follow-up after counseling. Six women had completed their family plans and another six did not wish to become mothers. Five women had a scheduled COS but drop out before the planned cycle. Five women did not want to perform COS but they did not give their reason to the practitioner. Four women had a spontaneous pregnancy. Three women had undergone emergency fertility preservation before fertility-impairing treatments and did not wish to undergo additional COS. One woman did not want to perform a COS for religious reasons and another one did not want hormonal treatment.

**Figure 1:**
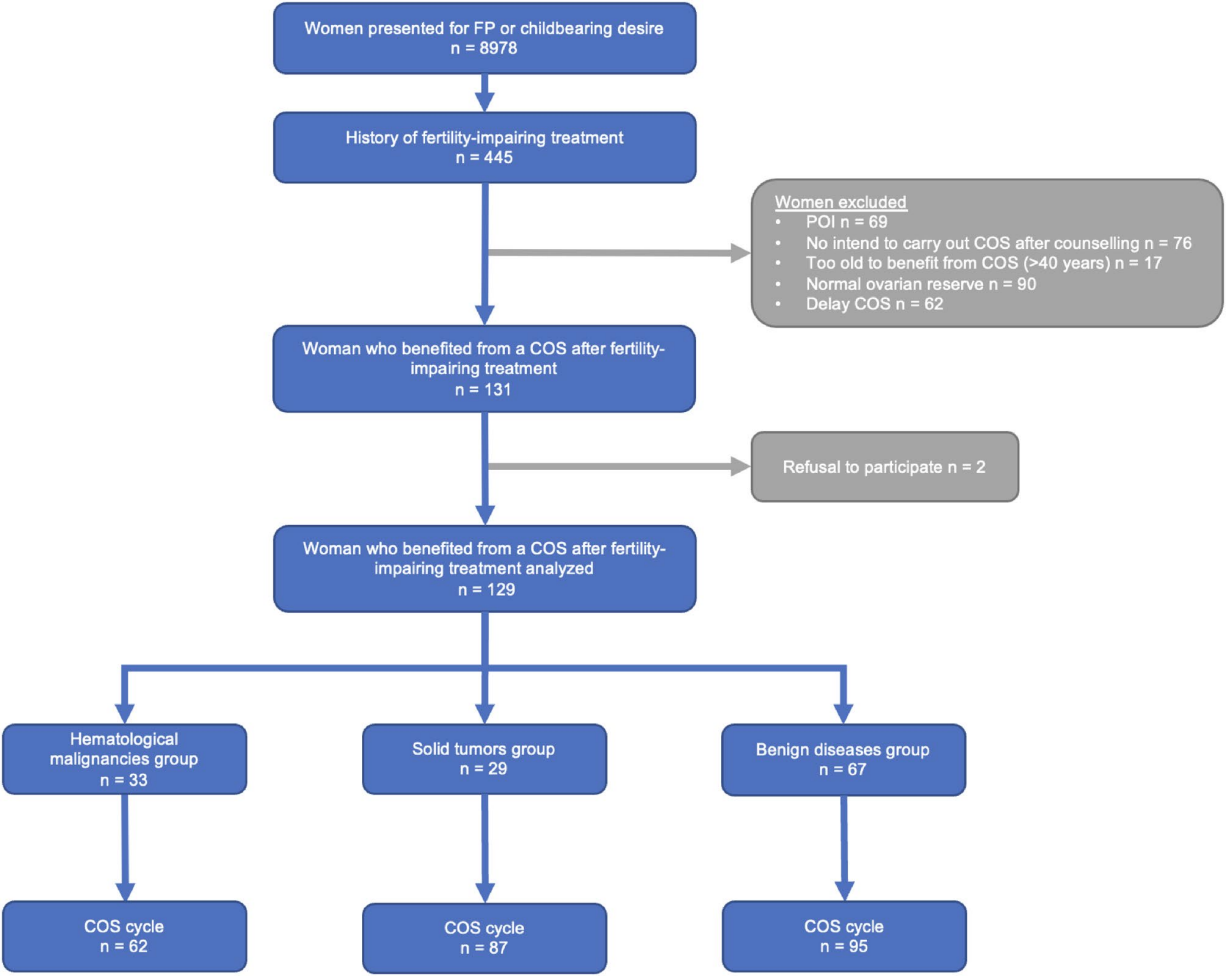
Fertility preservation after fertility-impairing treatment: distribution of initial diseases according to groups.

Finally, a total of 129 women were included in the study. Among these 129 women, 113 started COS for metaphase II oocyte cryopreservation and/or 25 started COS for metaphase II oocyte fertilization and fresh embryo transfer because of an immediate pregnancy plan. We studied a total of 244 started COS cycles: 200 COS cycles for FP and 44 COS cycles for IVF-ICSI with fresh embryo transfer (Fig. 1). Among these 129 women, metaphase II oocytes were obtained for 111 (86%) women and in 19 women (14%), no metaphase II oocytes were obtained (COS cancellation for absence of ovarian response or metaphase II oocyte retrieval failure). Three groups of women were composed as previously described: 33 in the hematological malignancy group (including 7 hematopoietic stem cell transplantations), 29 in the solid tumor group, and 67 in the benign disease group. The baseline characteristics of the 3 groups of women are presented in Table 1. Regarding the initial characteristics of the women, age at initial diagnosis was significantly lower in the hematological malignancy group (20.5 ± 8.0 years) than in the solid tumor group (24.1 ± 9.0 years) and benign disease group (25.4 ± 6.7 years) (p = 0.03). The AMH median levels were 1.4 ± 1.8, 2.5 ± 3.2 and 1.7 ± 1 ng/ml for the hematological malignancy group, solid tumor group and benign disease group, respectively (ng/ml ± interquartile range).

**Table 1:** Characteristics of women who started controlled ovarian stimulation after fertility-impairing treatment and women’s results of controlled ovarian stimulation after fertility-impairing treatment:

	Hematological malignancies group (n = 33)	Solid tumors group (n = 29)	Benign diseases group (n = 67)	p-value
Age at diagnosis (years)	20.5 ± 8.0	24.1 ± 9.0	25.4 ± 6.7	0.03
Age at first COS (years)	25.9 ± 6.1	28.6 ± 6.7	28.9 ± 5.8	NS
BMI (kg/m^2^)	21.2 ± 3.0	22.2 ± 4.3	23.4 ± 4.4	0.03
Gestity	0.3 ± 0.8	0.2 ± 0.6	0.4 ± 0.8	NS
Parity	0.2 ± 0.5	0.1 ± 0.4	0.1 ± 0.3	NS
Smoking > 10 cigarettes/day	0 (0.0)	1 (3.6)	8 (11.9)	NS
AMH (ng/ml)	1.4 ± 1.8	2.5 ± 3.2	1.7 ± 1.8	NS
FSH (IU/L)	14.1 ± 24.8	9.2 ± 3.9	7.3 ± 2.5	NS
AFC	9.7 ± 8.0	12.5 ± 8.2	10.0 ± 6.7	NS
No history of FP before fertility-impairing treatment	27 (75.0)	26 (92.9)	60 (89.6)	NS
No. of cycle with metaphase II oocyte retrieval	22 (61.1)	25 (89.3)	64 (95.5)	< 0.01
No. of cycle with metaphase II oocyte retrieval failure	1 (2.8)	1 (3.6)	1 (1.5)	NS
No. of cycle with cancellation for absence of ovarian response	11 (30.6)	3 (10.7)	2 (3.0)	< 0.01
No. of metaphase II oocyte vitrification cycle	27 (75.0)	23 (82.1)	63 (94.0)	NS
No. of fresh IVF cycle	5 (13.9)	8 (28.6)	4 (6.0)	0.02
No. of fresh ICSI cycle	1 (2.8)	1 (3.6)	6 (9.0)	NS
No. of metaphase II oocyte thawing cycle	1 (2.8)	2 (7.1)	3 (4.5)	NS
No. of frozen embryo transfer cycle	2 (5.6)	4 (14.3)	3 (4.5)	NS
No. of COS cycles per woman	1.8 ± 1.0	1.8 ± 1.4	1.8 ± 1.1	NS
Total no. of metaphase II oocyte retrieved per woman	10.7 ± 4.6	12.3 ± 9.1	10.1 ± 7.6	NS
Spontaneous pregnancy	2 (5.6)	0 (0.0)	2 (3.0)	NS
No. of metaphase II oocytes fertilization cycle	4 (11.1)	5 (17.9)	8 (11.9)	NS
No. of metaphase II oocytes vitrified return to use cycle	1 (2.8)	1 (3.6)	2 (3.0)	NS
No. of fresh metaphase II oocytes use cycle	3 (8.3)	6 (21.4)	6 (9.0)	NS

Data are expressed as the mean ± SD or *n* (%) if not specified. Median are expressed with [1st quartile- 3rd quartile].

*COS* controlled ovarian stimulation, *BMI* body mass index, *AMH* anti-Müllerian hormone, *FSH* follicle stimulating hormone, *AFC* antral follicle count on day 3, *FP* fertility preservation, *IVF* in vitro fertilization, *ICSI* intracytoplasmic sperm injection, *FP* fertility preservation.

Women's results of COS after fertility-impairing treatment are presented in Table 1. The total cumulative number of metaphase II oocytes collected per woman who started COS was similar in the hematological malignancy group (mean of 10.7 ± 4.6 metaphase II oocytes collected), the solid tumor group (mean of 12.3 ± 9.1 metaphase II oocytes collected) and benign disease group (mean of 10.1 ± 7.6 metaphase II oocytes collected) (p = 0.46).

The total number of started COS cycles performed per woman was similar in the hematological malignancy group (1.8 ± 1.0), the solid tumor group (1.8 ± 1.4) and the benign disease group (1.8 ± 1.1) (p = 0.99).

The number of women for whom a COS cycle cancellation for absence of ovarian response had to be decided was significantly higher in the hematological malignancy group (n = 11; 30.6%) than in the solid tumor group (n = 3; 10.7%) and benign disease group (n = 2; 3%) (p < 0.01).

The number of women who presented an oocyte retrieval failure was higher in the solid tumor group (n = 1; 3.6%) than the hematological malignancy group (n = 1; 2.8%), and the benign disease group (n = 1; 1.5%) (p < 0.01).

The results of ovarian stimulation per started cycle after fertility-impairing treatment are presented in Table 2. The antagonist protocol was used in 80.4% of the COS cycles (n = 197). The mean number of metaphase II oocytes collected per started COS cycle was similar in the hematological malignancy group (5.5 ± 3.3), the solid tumor group (5.5 ± 4.6) and the benign disease group (5.9 ± 4.4) (NS).

**Table 2:** Results per started cycle of controlled ovarian stimulation fertility-impairing treatment.

	Hematological malignancies group (n = 62)	Solid tumors group (n = 87)	Benign diseases group (n = 95)	p-value
No. of cycles cancellation for absence of ovarian response	16 (25.8)	6 (6.9)	5 (5.3)	< 0.01
No. of metaphase II oocyte retrieval failure	2 (3.2)	8 (9.2)	2 (2.1)	NS
Type of stimulation				
Metaphase II oocyte vitrification	54 (87.1)	61 (70.1)	85 (89.5)	< 0.01
IVF	7 (11.3)	20 (23)	4 (4.2)	< 0.01
ICSI	1(1.6)	6 (6.9)	6 (6.3)	NS
Type of stimulation protocol				
Antagonist	61 (98.4)	61 (70.1)	75 (78.9)	< 0.01
Random start	1 (1.6)	3 (3.4)	1 (1.0)	NS
Short	0 (0.0)	10 (11.5)	0 (0.0)	0.04
Progesterone block protocol	0 (0.0)	3 (3.4)	7 (7.4)	NS
Long agonist	0 (0.0)	9 (10.3)	10 (10.5)	< 0.01
Daily long agonist	0 (0.0)	0 (0.0)	2 (2.1)	NS
Natural cycle	0 (0.0)	1 (1.1)	0 (0.0)	NS
Length of COS (days)	9.8 ± 1.8	9.9 ± 2.5	10.1 ± 2.5	NS
Total gonadotropin dose (IU)	3444.8 ± 963.4	3250.4 ± 1381.1	3439.1 ± 1207.1	< 0.01
Triggering type				
HCG trigger	15 (24.2)	31 (35.6)	27 (28.4)	NS
GnRHa trigger	12 (19.4)	16 (18.4)	14 (14.7)	NS
HCG + GnRHa trigger	18 (29.0)	34 (39.1)	6.8 (3.6)	< 0.01
No. of mature follicles at trigger day	6.6 ± 3.1	6.7 ± 4.6	6.8 ± 3.6	NS
E2 at trigger day (pg/ml)	1182.7 ± 768.7	1868.1 ± 2078.1	1264.8 ± 1127.2	< 0.01
No. of metaphase II oocyte retrieved per COS	5.5 ± 3.3	5.5 ± 4.6	5.9 ± 4.4	NS
Total use of metaphase II oocytes	5 (3.2)	32 (36.0)	11 (11.6)	NS
No. of metaphase II oocytes vitrified return to use cycle	1 (1.6)	10 (11.5)	1 (1.1)	NS
No. of fresh metaphase II oocytes use cycle	4 (6.5)	24 (27.6)	9 (9.5)	< 0.01

Data are expressed as the mean ± SD or *n* (%) if not specified. Median are expressed with [1st quartile- 3rd quartile].

*COS* controlled ovarian stimulation, *IVF* in vitro fertilization, *ICSI* intracytoplasmic sperm injection, *HCG* human chorionic gonadotropin, *GnRHa* antagonist gonadotropin releasing hormone, *E2* estradiol.

The fertility outcome results after delayed COS post-impaired fertility treatment followed by fertilization are presented in Table 3. The women had an average follow-up of 78.7 ± 75.73 months.

**Table 3:** Fertility outcome of controlled ovarian stimulation after fertility-impairing treatment in women who planned a pregnancy.

	Hematological malignancies group (n = 5)	Solid tumors group (n = 26)	Benign diseases group (n = 10)	p-value
Age at embryo transfer (years)	36.3 ± 4.6	33.1 ± 2.8	31.7 ± 3.4	NS
Average follow-up after PF (months)	94.3 ± 79.3	82.0 ± 86.4	63.9 ± 61.3	NS
Type of stimulation				
Fresh IVF	4 (14.7)	16 (61.5)	4 (40.0)	< 0.01
Fresh ICSI	0 (0.0)	5 (19.2)	5 (50.0)	< 0.01
No. of cycle of metaphase II oocyte thawing	1 (20.0)	5 (19.2)	1 (10.0)	NS
Fertilization rate by metaphase II oocyte	77.6 ± 21.9	66.7 ± 30.7	53.5 ± 41.9	NS
No. of diploid embryos	4.2 ± 1.6	3.0 ± 2.2	2.7 ± 3.2	0.01
No. of cryopreserved embryos	2.2 ± 1.9	0.8 ± 1.9	1.4 ± 2.7	NS
No. of fresh embryos transferred	1.6 ± 0.9	1.2 ± 1.0	0.9 ± 0.9	NS
No. of frozen embryos transferred	0.8 ± 1.3	0.7 ± 1.1	0.5 ± 1.3	NS
Cumulative pregnancy outcomes				
Cumulative biochemical pregnancy rate	4 (80.0)	8 (30.8)	4 (80.0)	NS
Cumulative clinical pregnancy rate	1 (20.0)	8 (30.8)	2 (20.0)	NS
Cumulative live birth rate	1 (20.0)	5 (19.2)	2 (20.0)	NS

Cumulative results per cycle of controlled ovarian stimulation followed by fertilization.

Data are expressed as the mean ± SD or *n* (%) if not specified. Median are expressed with [1st quartile- 3rd quartile].

*FP* fertility preservation, *IVF* in vitro fertilization, *ICSI* intracytoplasmic sperm injection.

Among a total of 1222 vitrified metaphase II oocytes, 202 (16.5%) metaphase II oocytes were thawed and fertilized in 21 women who wanted to conceive. The CLBR per stimulation cycle, including fresh and frozen embryo transfers from thawed or fresh metaphase II oocytes, was similar in the hematological malignancy group (n = 1; 20%), the solid tumor group (n = 5; 19.2%) and benign disease group (n = 2; 20%) (NS), with a total of 8 live births. In the subgroup of women who wanted to conceive, the cumulative live birth rate per woman was 38%, with 8 live births for these 21 women.

## Discussion

We evaluated a large cohort of women who were offered a systematic strategy systematic delayed FP strategy after fertility-impairing treatment for both cancer and benign diseases.

With the cumulative number of metaphase II oocytes collected as the main objective, regardless of their subsequent use or the subsequent origin of the disease, we have chosen to place ourselves from the woman's perspective. Comparing all types of women and all types of disease allows us to refocus on the initial goal of fertility preservation and assisted reproductive technology (ART): to give women a real chance of pregnancy.

In our study, no difference was observed between the groups concerning the number of metaphase II metaphase II oocytes retrieved, either per women who started COS cycle or per started COS cycle, irrespective of the initial indication of FP. The number of metaphase II oocytes retrieved per woman is in agreement with Chan et al., who recovered an overall average of 8 (CI 2–19) metaphase II oocytes per woman after chemotherapy exposure for cancer in 130 women^21^. Legrand et al. reported an average of 6.4 ± 3.4 retrieved metaphase II oocytes in 70 women with benign ovarian tumors with a history of previous surgery and multiple or large cyst indications^22^. These results are also in agreement with Volodarsky-Perel et al., who reported an average of 10 cryopreserved metaphase II oocytes per woman before gonadotoxic treatments for cancer, regardless of the cancer grade^23^. The fertilization rate after fertility-impairing treatment observed our study is also encouraging and in agreement with that found by Kato et al.^24^. With an overall vitrified-warmed metaphase II oocyte to live born child efficiency rate of 6.4%, the mean of 10 cryopreserved metaphase II oocytes per woman gives these women real opportunities for live birth^25^. In a large study by Cobo et al., when FP was performed before the age of 35 and for malignant disease, the CLBR was 9.1% (− 0.7 to 19) for 5 metaphase II oocytes, 35.8% (14.3–57.2) for 8 metaphase II oocytes and 42.9% (19.7–66.1) for 10 metaphase II oocytes. They concluded that with ~ 25 metaphase II oocytes, the CLBR probability rises to ~ 95%.

The total of 8 pregnancies achieved in our study led to a 20% live birth rate per stimulation cycle with metaphase II oocyte fertilization, either vitrified-warmed or fresh.

As the CLBR per metaphase II oocyte decreases with age, rapid referral after every fertility-impairing treatment should be supported as soon as possible and before the age of 35 years^25^. In a retrospective observational study including 485 women with endometriosis, Cobo et al. reported that the CLBR increased with the number of metaphase II oocytes per woman, reaching 89.5% (95% CI = 80.0–99.1) for 22 thawed metaphase II oocytes. In the group aged ≤ 35 years, the CLBR was 95.4% (95% CI = 87.2–103.6) using ~ 20 metaphase II oocytes compared with that of 79.6% (95% CI = 58.1–101.1) for women aged > 35 years (P < 0.05)^26^.

In the long-term after cancer, distress about interrupted childbearing persists, particularly in women without children^12^. Among cancer survivors, the negative impact on quality of life due to reproductive concerns and unmet information needs when making fertility decisions about creating a future family has been reported^27^. Deshpande et al. reported that pretreatment fertility counseling leads to lower levels of posttherapy regret and better quality of life, suggesting that posttreatment counseling may be similarly beneficial^28^. Routinely offering FP counseling to all women after fertility-impairing treatment could improve their overall quality of life, regardless of whether the consultation leads to delayed metaphase II oocyte cryopreservation.

Offering a systematic delayed FP strategy after chemotherapy and before the occurrence of POI should be easy to systematize. The setup of oncofertility networks and platforms allows for systematic phone calls to women 12 months after their last treatment^29^.

An increasing number of authors are focusing their research on FP for nonmalignant diseases^4^. Legrand et al. reported that metaphase II oocyte banking in women treated for benign ovarian tumors is an efficient strategy, with a mean of 7.0 (± 5.23 SD) cryopreserved metaphase II oocytes per woman, irrespective of the histological type of tumor^22^. Concerning endometriosis, Cobo et al. reported a mean number of cryopreserved metaphase II oocytes of 8.5 (± 4.8 SD) per woman for those with a history of unilateral surgery and 8.0 (± 5.7 SD) per woman for those with a history of bilateral surgery. The CLBRs were 40.4% and 49.3%, respectively, with a total of 101 babies born^30^. When focused on age and history of surgery, they observed a CLBR of 52.8% for women under 35 years old and 29.3% for women over 35 years old.

No study has evaluated the benefit of FP on quality of life in women with benign diseases or on the cost-effectiveness balance. Further studies are required to explore these aspects, but given the data on oncofertility, we hypothesize that delayed FP could be beneficial for improving the quality of life of women with decreased ovarian reserve after fertility-impairing treatment.

Some limitations of our study must be discussed. First, the retrospective nature of our study and the low metaphase II oocyte use rates (16.5%) make it difficult to interpret the results of the CLBR. The large number of women who did not wish to undergo metaphase II oocyte cryopreservation despite an obvious indication must also be considered. The French insurance coverage for fertility preservation also raises the problem of extrapolation to countries where woman have to pay for fertility preservation.

The main result of our study is the feasibility of metaphase II oocyte banking in women with DOR after fertility-impairing treatment with a cumulative number of cryopreserved metaphase II oocytes between 10.1 (± 7.6 SD) and 12.3 (± 9.1 SD) regardless of the initial disease. The CLBR of 20% per cycle is also important data to provide when counseling referred women.

Regardless of the initial disease that led to DOR after fertility-impairing treatment, metaphase II oocyte banking should be offered to all reproductive-age women under 35 years of age after every fertility-impairing treatment. The overall improvement in access to FP prior to and after fertility-impairing treatment must be continued through the education of practitioners and throughout the setup of fertility networks^13^.

## Data Availability

Individual participant data are available on 10.5281/zenodo.6363664.
